# Brassicasterol inhibits hepatitis B virus-associated hepatocellular carcinoma development via suppression of AKT signaling pathway

**DOI:** 10.1186/s13027-023-00502-1

**Published:** 2023-04-20

**Authors:** Jindi Zeng, Jiancheng Wu, Shuijiao Pang, Feifei Wang, Xin Yu, Shouhua Zhang, Junquan Zeng, Jinlong Yan, Jianping Lian

**Affiliations:** 1 Department of Pharmacy, The Affiliated Hospital of Jinggangshan University, Ji’an, 343000 Jiangxi Province China; 2 Department of General Surgery, Second Affiliated Hospital of Nanchang University, No1 Minde Road, Nanchang, 330006 Jiangxi Province China; 3 Department of General Surgery, Jiangxi Provincial Children’s Hospital, Nanchang, 330006 Jiangxi Province China; 4 Department of Oncology, The Affiliated Hospital of Jinggangshan University, Ji’an, 343000 Jiangxi Province China

**Keywords:** Brassicasterol, Hepatitis B virus, Inhibition, Hepatocellular carcinoma

## Abstract

**Background:**

Hepatitis B virus (HBV)-associated hepatocellular carcinoma (HCC) does not respond well to current treatment options like sorafenib, and there is an urgent need for developing therapeutical strategies for HBV + HCC. Brassicasterol has previously shown anti-cancer and anti-viral activities, however, its value against HBV + HCC remains to be explored.

**Methods:**

The inhibitory effect of brassicasterol and sorafenib was evaluated on HBV + HCC cell lines and xenograft mouse model. The cytotoxicity of brassicasterol on normal liver cells were measured by LDH assay. AKT agonist was used to identify the targeted signaling pathway by brassicasterol.

**Results:**

Brassicasterol induced HBV + HCC cell death in a both dose-dependent and time-dependent manner, and such inhibition was more potent than sorafenib. Brassicasterol did not show apparent cytotoxicity to normal liver cells. Xenograft mouse model further confirmed the inhibitory effect of brassicasterol on the growth of HBV + HCC. Furthermore, signaling pathway analysis showed that brassicasterol-treated HBV + HCC cells had decreased level of phosphor-AKT expression while the addition of AKT agonist could counteract the inhibitory effect of brassicasterol on HCC, indicating that brassicasterol suppressed AKT pathway to exhibit anti-cancer activity in HBV + HCC cells. In addition, brassicasterol showed similar levels of inhibition on HBV− and HBV + HCC cells.

**Conclusion:**

Brassicasterol possesses anti-cancer activity against HCC through the downregulation of AKT pathway and such activity is independent of HBV infection.

**Supplementary Information:**

The online version contains supplementary material available at 10.1186/s13027-023-00502-1.

## Background

As one of the most prevalent cancers worldwide, hepatocellular carcinoma (HCC) is a leading cause of cancer-related death, accounting for more than 780,000 deaths every year [1, 2]. Various risk factors are associated with HCC, but hepatitis B virus (HBV) infection tops the ranking list and are estimated to contribute to more than 60% of all HCC cases worldwide [2, 3]. HBV infection is prevalent around the globe. Although vaccination against HBV has greatly reduced the infection rate, there are still 5–10% of the population that remains unprotected due to waned immunity over time [4]. In addition, the limited vaccination coverage is accountable for a major portion of new HBV infections [5]. Currently, approximately 257 million people are living with chronic HBV infection and 25 – 40% % of them have the lifetime risk of developing adverse outcomes HCC or cirrhosis [6, 7].

Currently, the common treatment options for early-stage HCC are still surgical resection, liver transplantation and radiofrequency ablation [8, 9]. However, in HBV + HCC patients, the recurrence rate is high after such treatments [1, 9]. The treatment options for late-stage HCC are very limited, because conventional systemic chemotherapy offers unsatisfactory benefit [8]. The main options for late-stage HCC patients are multikinase inhibitors like sorafenib, lenvatinib and regorafenib etc. [8–10]. Sorafenib, a multikinase inhibitor, can suppress cancer cell growth by promoting apoptosis and mitigating angiogenesis [11]. Although survival benefits have been observed in HCC patients after treatment with these drugs, the responses are not usually durable because of the rapid developed drug resistance [9, 12]. Moreover, it has been revealed in several clinical trials that HBV + HCC patients do not respond well to multikinase inhibitors like sorafenib comparing to patients without HBV infection [13, 14]. Therefore, there is an urgent need for developing novel therapeutical strategies for HBV + HCC.

Brassicasterol (24-methyl cholest-5,22-dien-3β-ol; C28H46O) is a type of phytosterol mainly produced by unicellular algae and some terrestrial plants like rape [15]. This natural product has been widely used as a food additive and is known for its cardiological health benefits [16, 17]. Recently, bassicasterol has been reported to show anti-cancer and anti-viral activities [18–21]. However, its value in treatment for HCC, especially HBV + HCC, has not yet been investigated.

In the current study, we investigated the therapeutic value of brassicasterol against HBV + HCC both in vitro and on a xenograft mouse model. We also further explored the mechanism of action of brassicasterol against HBV + HCC.

## Materials and methods

### Cells and drugs

Cell lines HepG2.215, PLC5, HepG2, LO2, AML12, and FL83B were all purchased from the Type Culture Collection of the Chinese Academy of Sciences (Shanghai). HepG2.215 and PLC5 are both HBV-positive (termed as HBV + hereafter) HCC cell lines. HepG2.215 is a stably transfected with HBV, while PLC5 only secretes HBsAg but does not support HBV replication. HepG2 is an HBV-negative (termed as HBV- hereafter) HCC cell line. LO2, AML12, and FL83B are normal immortalized hepatocytes. LO2 is human origin while AML12 and FL83B are mouse origin.

HepG2, HepG2.215, PLC5 and LO2 were cultured in high glucose DMEM (Sangon) supplemented with 10% FBS (Hyclone) and penicillin (100 U/ml) and streptomycin (100 µg/ml) (Sangon). AML12 cells were cultured in DMEM/F12 medium (Sangon) supplemented with 10% FBS, 10 µg/mL insulin (ThermoFisher Scientific), 5.5 µg/mL transferrin (ThermoFisher Scientific), 5 ng/mL sodium selenite (ThermoFisher Scientific), 40 ng/mL (100 nM) dexamethasone (ThermoFisher Scientific) and antibiotics. FL83B cells were cultured in F-12K medium (Procell) supplemented with 10% FBS and antibiotics. Brassicasterol (cat no: B4936) was purchased from Sigma-Aldrich, Merck and sorafenib (cat no: sc-220125) was purchased from Santa Cruz and AKT agonist IGF-1 (cat no: ab9573) was purchased from Abcam.

### Cell culture and drug treatment

Cells were preseeded at 4 × 10^3^ cells/well into 96 well plates 16–18 h before drug treatment, and drugs were introduced by replacing old cell culture medium with fresh medium supplemented with drugs at desired concentrations. For drug dose response tests, cells were treated with sorafenib or brassicasterol at 0, 5, 10, 25 and 50 µM (HCC cells) or 0, 5, 10, 20, 40, 80, 160, 320, 640 and 1280 µM (normal liver cells) for 48 h before cell viability or cytotoxicity was determined. For drug time course response tests, cells were treated with 10 µM sorafenib or brassicasterol for up to 96 h, before cell viability or cytotoxicity was determined. For AKT agonist treatment, AKT agonist IGF-1 was introduced into the cell culture 6 h before the treatment of brassicasterol and remained in the culture till the cell viability assay.

### Cell viability assay

MTT assay was used to assess cell viability in this study using a commercial MTT kit (Abcam), as previously described with modifications [1]. In brief, cells in 96 well plates were first treated as described above, and then cell culture medium was removed and 100 µl of MTT reagent and serum-free medium at 1:1 ratio into each well. The plates were then incubated at 37 °C for 3 h. After incubation, 150 µl/well of MTT solvent was added and the plate was then shaken for 15 min on an orbital shaker at room temperature. Finally, plates were read at OD590 and cell viability was calculated with cells without drug treatment considered 100% viable.

### Cytotoxicity assay

LDH assay was used to assess drug cytotoxicity in this study with a commercial LDH assay kit (Abcam) following the manufacturer’s instructions. In brief, cells in 96 well plates were first treated as described above, and then plate gently shaken on an orbital shaker for 5 min to ensure even distribution of LDH. After shaking, plates were centrifuged to pellet cells and 10 µl/well of the clear medium were transferred to a new 96 plate, mixed with 100 µl/well LDH reaction mix. The plates were then incubated for 30 min at room temperature and then read at OD450 (testing wavelength) and OD650 (reference wavelength).

### Ethical statement

All experimental protocols involving animals were reviewed and approved by the Ethical Review Committee of the Affiliated Hospital of Jinggangshan University (Approval Number: LUNLI-2022-002) and performed in accordance with the local regulations.

### Tumor cell injection and drug treatment

The xenograft HCC mouse study was reviewed and approved by the institutional ethical review committee and performed as previously described with modifications [1]. In brief, HepG2.215 cells were first washed and resuspended in PBS at 2 × 10^7^ cells/ml, and then injected 300 µl cell suspension under the skin at the left flank. When the tumor volume reached 100 mm^3^, mice were treated intraperitoneally with brassicasterol or sorafenib both at 100 mg/kg every day for 15 days. For mock treatment, mice received equal volume of the same solvent as the drug treatment group. Tumor size and mouse weight were measured every 3 days. Tumor volume was calculated using ${(mean diameter)}^{3}\times \frac{\pi }{6}$. Tumors were also weighed at the time of animal sacrifice.

### Western blot

Western blot was performed as previously described with modifications [22]. In brief, cells were first mock treated or treated with brassicasterol for 48 h, and then cells were lysed with Pierce IP lysis buffer (Thermo Fisher Scientific) supplemented with Protease phosphatase inhibitors (Cell Signalling Technology). Centrifugation cleared cell lysates were then mixed with loading buffer and loaded onto an SDS-PAGE gel. After electrophoresis, the gel separated proteins were then transferred onto a PVDF membrane. The membrane was then sequentially blocked in 5% non-fat milk for 1 h at room temperature, and primary antibodies over night at 4 °C and HRP-conjugated secondary antibodies for 1 h at room temperature, respectively. Following extensive washes with PBST, the membrane was incubated with ECL Plus Western Blotting Substrate (BosterBio) and immuno-bands were imaged by a CCD camera. Semiquantification of the bands were achieved by Image J (Version 1.8.0; National Institute of Health, USA). The following primary antibodies were used: Rabbit anti-cleaved Caspase 3 (ab2302; Abcam), mouse anti-AKT antibody (60203-2-Ig; ProteinTech), rabbit anti-p-AKT antibody (4060; Cell Signalling Technology), rabbit anti-ERK1/2 antibody (51068-1-AP; ProteinTech), rabbit anti-p-ERK1/2 antibody (9101; Cell Signalling Technology) and mouse anti-GAPDH antibody (60004-1-Ig; ProteinTech). The following secondary antibodies were used: HRP-conjugated goat anti-rabbit IgG(H + L) (SA00001-2; ProteinTech) and HRP-conjugated goat anti-mouse IgG(H + L) (SA00001-1; ProteinTech).

### Statistical analysis

All data were expressed as mean ± standard deviation (SD). Kruskal–Wallis test followed by Dunn’s multiple comparisons test were used to identify the statistical significance between groups and *p* value less than 0.05 was considered statistically significant. All statistical analyses were performed with GraphPad Prism (version 9.4.0).

## Results

### The inhibitory effect of brassicasterol on HBV + HCC cells

**Fig. 1:**
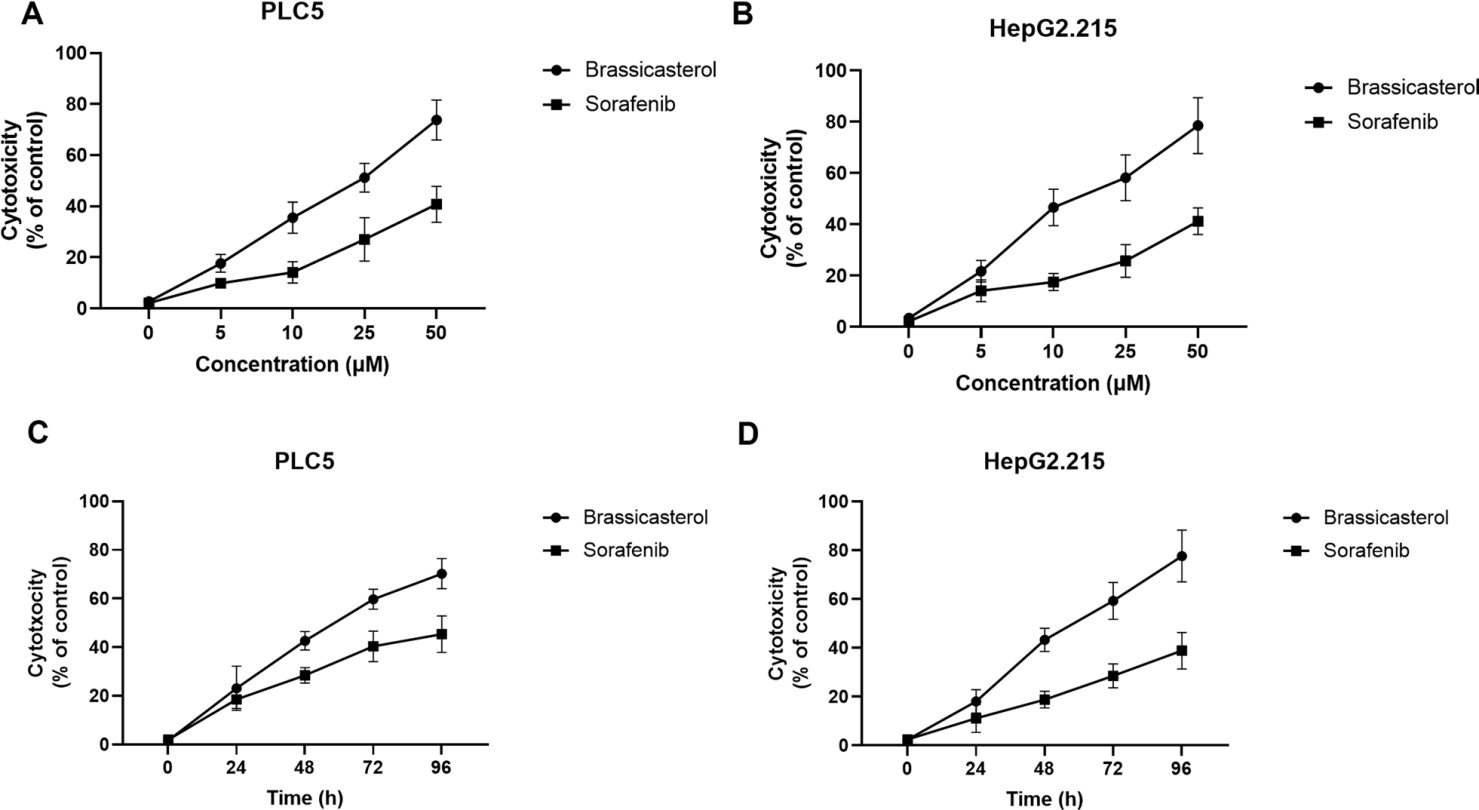
Brassicasterol promotes HBV + HCC cell cytotoxicity. **A** PLC5 and **B** HepG2.215 cells were incubated with increasing doses of brassicasterol or sorafenib for 48 h and cytotoxicity was assessed by LDH assay. **C** PLC5 and **D** HepG2.215 cells were incubated with 10 µM brassicasterol or sorafenib for up to 96 h and cytotoxicity was assessed by LDH assay. Data shown are mean ± SD of three independent experiments

To test the anti-cancer effect of brassicasterol on HBV + HCC, 2 HBV-associated HCC cell lines, PLC5 and HepG2.215, were used in the current study. The 2 cell lines were first treated with increasing doses of brassicasterol or the FDA-approved anti-HCC drug, sorafenib, for 48 h, and then cell viability was assessed. As shown in Fig. 1A–B and Figure S1A-B, both brassicasterol and sorafenib exhibited inhibitory effects on the viability of both HCC cell lines in a dose-dependent manner. Of note, brassicasterol induced much more cell death than sorafenib at the same dose, indicating that brassicasterol has more potent anti-cancer effect than sorafenib against HBV + HCC.

In addition to the dose–response evaluation, a time-course response for brassicasterol against HBV + HCC cells were also conducted. PLC5 and HepG2.215 cells were treated with 10 µM brassicasterol or sorafenib and cell viability was assessed at 0, 24, 48, 72 and 96 h. Similar to the dose–response evaluation, both drugs reduced viability of both HCC cell lines in an incubation time-dependent manner, with brassicasterol showing stronger impact on cell viability (Fig. 1C–D and Additional file 1: Fig. S1C–D).

Taken together, our data here indicate that brassicasterol has anti-cancer activity against HBV + HCC and its activity is stronger than FDA-approved drug sorafenib in vitro.

### Cytotoxicity of brassicasterol on normal liver cells

**Fig. 2:**
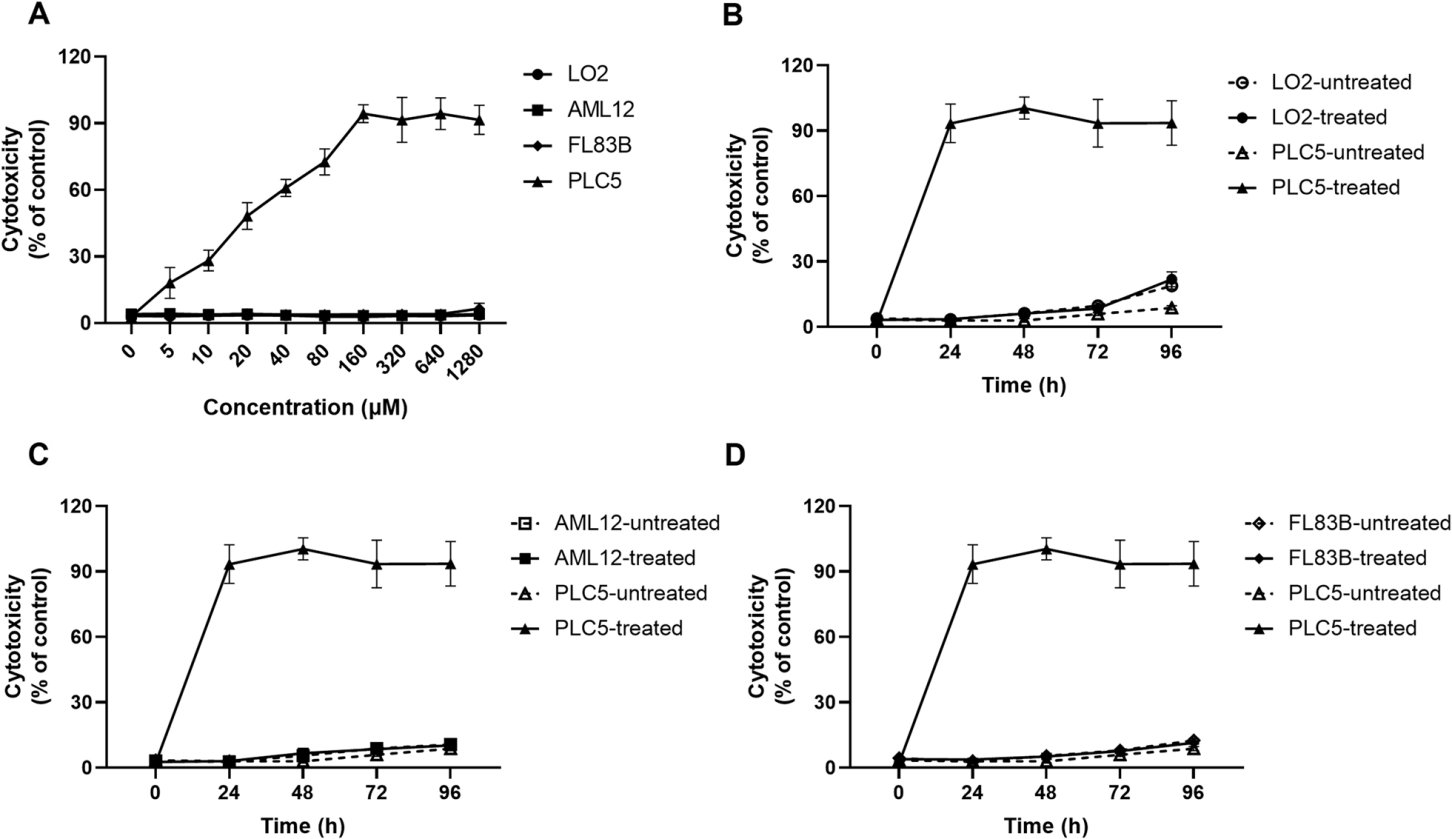
Brassicasterol has minimal cytotoxicity to normal liver cells. **A** Normal liver cell lines LO2, AML2 and FL83B cells together with control HCC cell line PLC5 were first incubated with various doses of brassicasterol for 48 h and then cell cytotoxicity was assessed by LDH assay. **B** Cells were incubated with 1000 µM brassicasterol for up to 96 h and cell cytotoxicity was assessed by LDH assay. Data shown are mean ± SD of three independent experiments

In general, an ideal anti-cancer drug should specifically kill the cancer cells without causing damage on healthy cells. To test the cytotoxicity of brassicasterol to normal liver cells, normal non-tumor hepatocyte cell lines LO2, AML2 and FL83B were used. Cells were first treated with increasing doses of brassicasterol for 48 h and then cytotoxicity was assessed by LDH assay (Fig. 2) and MTT assay (Additional file 1: Figure S2). Our data showed that a concentration up to 1280 µM of brassicasterol did not show apparent cytotoxicity to normal liver cells after 48 h incubation while a dose-dependent cell death was detected in HBV + HCC cell line PLC5 (Fig. 2A and Additional file 1: Fig. S2A). To further explore the effect of long incubation time of brassicasterol on the health of normal liver cells, these cell lines were incubated with 1000 µM brassicasterol for up to 96 h and cytotoxicity was assessed at 0, 24, 48, 72 and 96 h. As shown in Fig. 2B and S2B, all 3 normal liver cell lines did not show apparent cell death induced by brassicasterol, while HBV + HCC cell line PLC5 showed almost 100% cell death after 24 h of incubation with brassicasterol at this concentration. These data indicate that brassicasterol is not toxic to healthy normal liver cells.

### Brassicasterol suppresses the growth of HBV + HCC on the xenograft mouse model.

**Fig. 3:**
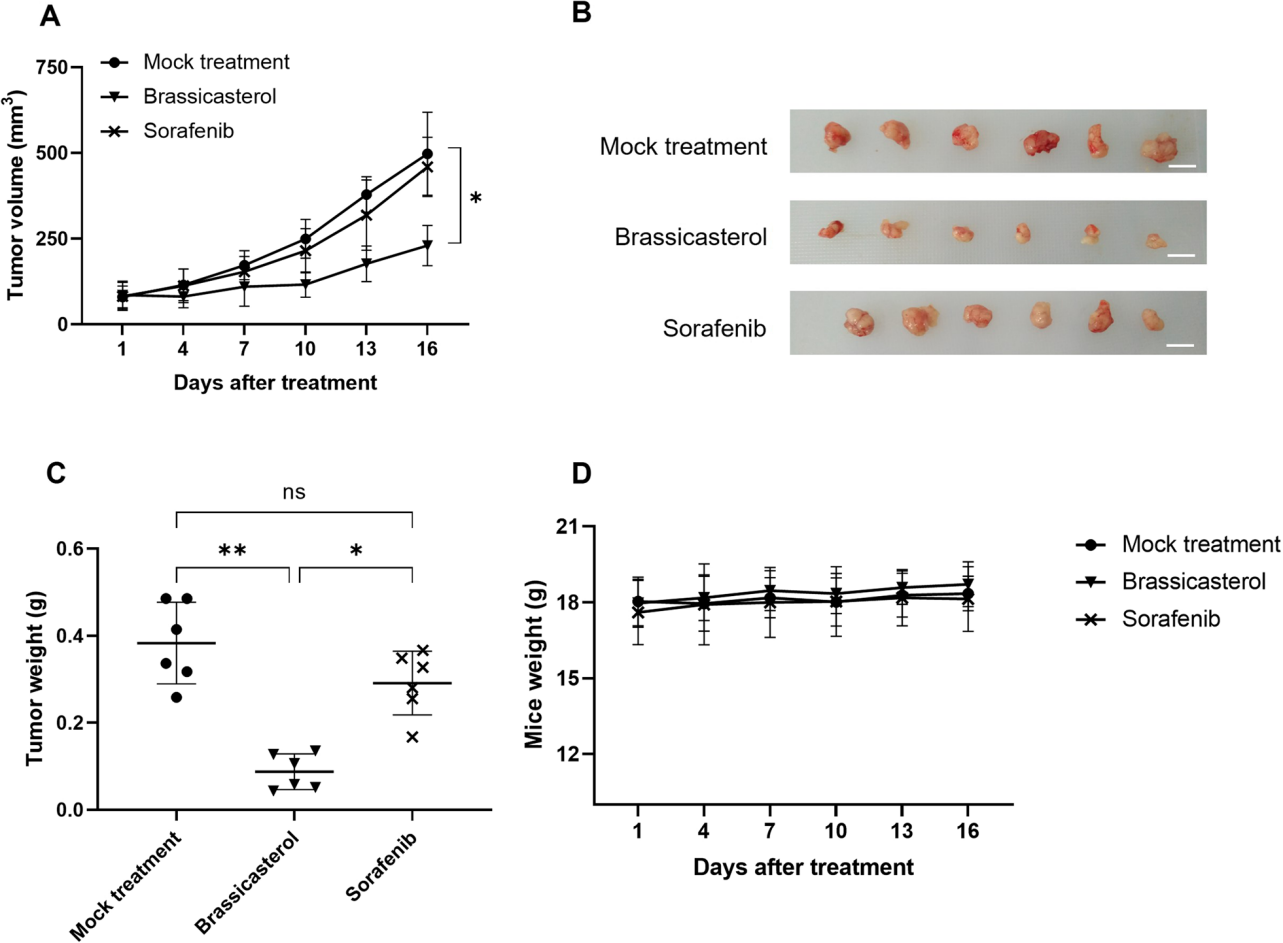
Brassicasterol inhibits HBV + HCC xenografted tumor growth in mice. Nude BLAB/c mice bearing xenografted HepG2.215-derived tumors were either mock-treated or treated with sorafenib or brassicasterol for 15 days. **A** Tumor volumes were measured every 3 days for up to 15 days since the treatment. ^*^, *p* < 0.05. **B** Tumor sizes and **C** tumor weights on the day of animal sacrifice (day 16) were shown. ns, not statistically significant; ^*^, *p* < 0.05; ^**^, *p* < 0.01. Scale bar: 1 cm. **D** Mice were weighed every 3 days for up to 15 days since the treatment

A study on a xenograft mouse model was also performed to further explore the therapeutic value of brassicasterol on HBV + HCC. Mice bearing xenografted HepG2.215 cell-derived tumor were either mock-treated, or treated with brassicasterol or sorafenib. As shown in Fig. 3A–B, sorafenib treatment also showed marginal effect on the inhibition of tumor growth, while brassicasterol significantly reduced the tumor volume. Similarly, tumor weight from mice treated with brassicasterol was significantly lower than that from mice received mock treatment or sorafenib treatment (Fig. 3C). Mice weight did not show any apparent change from all groups (Fig. 3D).

### Brassicasterol inhibits HBV + HCC growth via AKT pathway

**Fig. 4:**
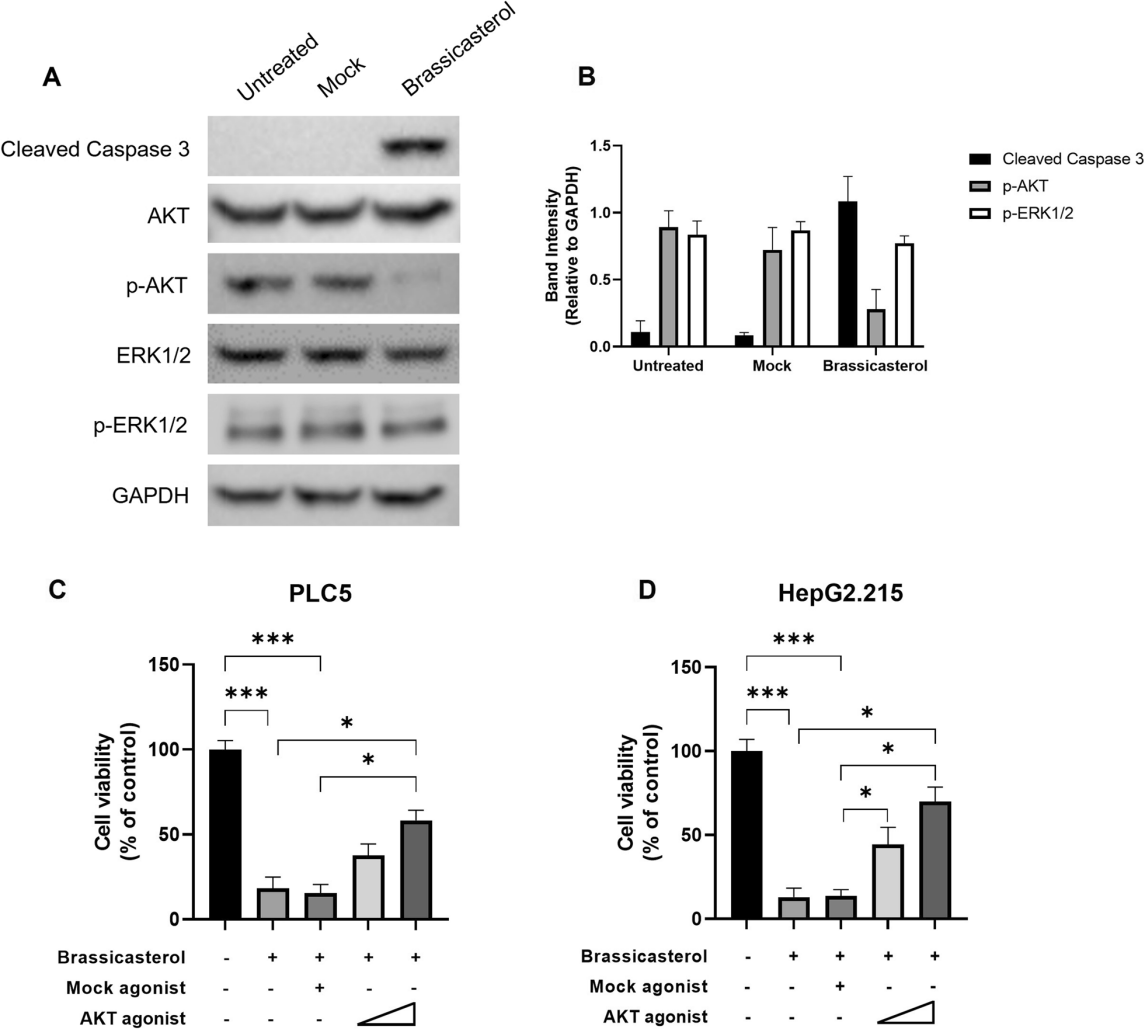
Brassicasterol inhibits HBV + HCC cell growth through AKT signaling pathway. **A**–**B** HepG2.215 cells were first either untreated, mock-treated or treated brassicasterol for 48 h and then the expression cleaved caspase 3, AKT, p-AKT, ERK1/2 and p-ERK1/2 was measured by western blot. **A** One representative data of three is shown. **B** Densitometry analysis of western blot data was performed with Image J. Data shown are mean ± SD of three independent experiments. **C** PLC5 and **D** HepG2.215 cells were either mock-treated or treated with brassicasterol in the presence or absence of AKT agonist for 48 h, and then cell viability was assessed by MTT assay. Data shown are mean ± SD of three independent experiments. ^*^, *p* < 0.05; ^**^, *p* < 0.01; ^***^, *p* < 0.001

AKT and ERK pathways are the common pathways involving tumorigenesis and targeting these pathways are proven to be effective against several cancers [23, 24]. In addition, previous study has shown that brassicasterol inhibited prostate cancer growth by targeting AKT pathway [19]. To investigate if brassicasterol suppressed the growth of HBV + HCC through downregulation of one or both pathways, western blot was performed. Our data showed that in HepG2.215 cells, p-AKT and p-ERK1/2 were expressed in high levels, indicating the activated AKT and ERK pathways (Fig. 4A–B). Upon brassicasterol treatment, the level of p-AKT, but not p-ERK1/2, was considerably reduced, together with increased expression of cleaved caspase 3, an indicator of cell apoptosis (Fig. 4A–B).

To further confirm that AKT pathway was targeted by brassicasterol in HCC inhibition, PLC5 and HepG2.215 cells were treated with brassicasterol in the presence or absence of AKT agonist. Our data showed that in the presence of AKT agonist, the anti-HCC effect was compromised in both cell lines (Fig. 4C–D). More importantly, the AKT agonist exhibited a dose-dependent effect in rescuing cell viability of both HCC cell lines (Fig. 4C–D). These data here indicate that brassicasterol inhibits HBV + HCC cell growth through suppression of the AKT pathway.

**Fig. 5:**
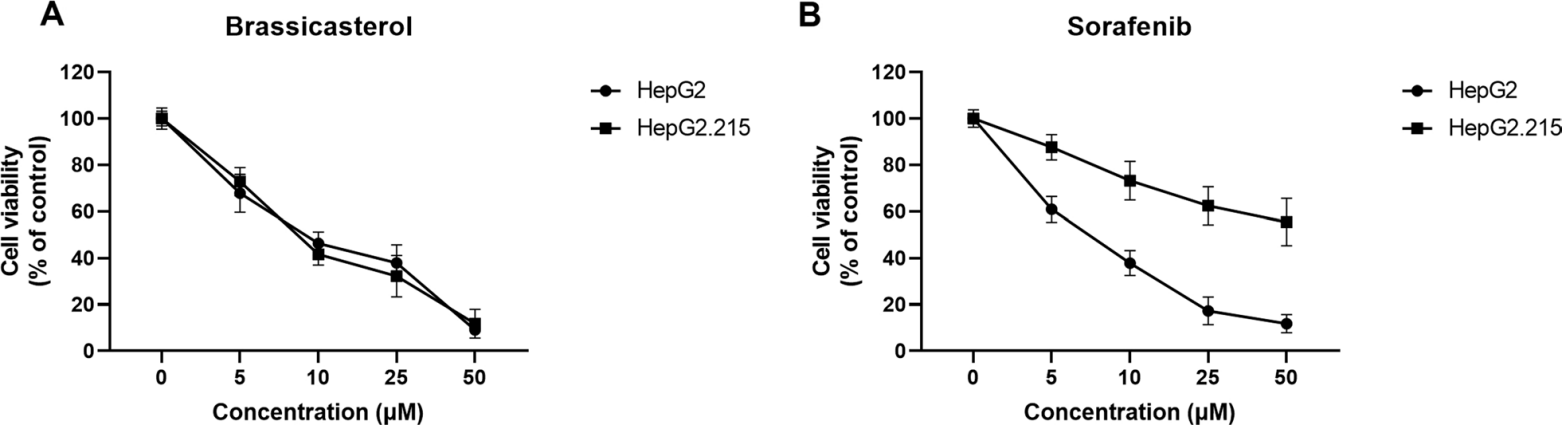
The anti-HCC effect of brassicasterol and sorafenib on HBV + and HBV- HCC cells. HBV- HCC cell line HepG2 and HBV + HCC cell line HepG2.215 were incubated with increasing doses of **A** brassicasterol or **B** sorafenib for 48 h and cell viability was assessed by MTT assay. Data shown are mean ± SD of three independent experiments

In addition, we also investigated whether the anti-HCC activity of brassicasterol was HBV infection dependent or independent. HepG2 (HBV-) and HepG2.215 (HBV+) cells were first treated with increasing doses of brassicasterol or sorafenib, and the cell viability was assessed. In agreement with previous findings, sorafenib induced considerably more cell death in HBV- HepG2 cells than HBV + HepG2.215 cells (Fig. 5). However, such difference was not observed in brassicasterol-treated cells, because equivalent levels of cell death was detected in both HepG2 and HepG2.215 cells (Fig. 5). These data indicate that the anticancer activity of brassicaseterol against HCC is HBV infection independent.

## Discussion

The development of curative treatment for HBV + HCC remains a challenge, due to several factors. First, although treatment options for HBV infections are available, there is still no cure [25]. Second, there is a high recurrence rate of neoplastic lesions for patients who received resection surgery [26, 27]. Third, HBV + HCC responds less well to current treatment options, like multikinase inhibitors [9, 12, 28]. Our previous studies have shown that HBV infection could promote HCC survival and also induce resistance to multikinase inhibitor sorafenib via AKT signaling pathway [1, 29]. Potentially, targeting the AKT signaling pathway could restrict HCC survival and reverse sorafenib resistance. In our current study, brassicasterol inhibited HCC growth both in vitro on cell lines and also in xenograft mouse model. And interestingly, the anti-HCC activity of brassicasterol was via the suppression of the AKT signaling pathway, indicating the importance of AKT pathway in HBV + HCC. In addition, our data showed that brassicasterol exhibited more potent activity against HBV + HCC than sorafenib. We also compared the anti-HCC activities of brassicasterol and sorafenib on both HBV- and HBV + HCC cells, and in agreement with previous findings, sorafenib showed much stronger inhibitory effect on HBV- HCC cell line HepG2 than HBV + HCC cell line HepG2.215 (Fig. 5). Interestingly, brassicasterol, unlike sorafenib, demonstrated comparable levels of inhibition on both HepG2 and HepG2.215 cells, indicating that the anti-HCC activity of brassicasterol is independent of HBV infection (Fig. 5). Although further studies are required to characterize the activity of brassicasetrol on HBV + HCC, brassicasterol may serve as a novel treatment option for patients with developed resistance to multikinase inhibitor drugs like sorafenib.

Brassicasterol has been long used as a food additive, however, its therapeutic values in various diseases have only been studied very recently. Brassicasterol has shown anti-viral and anti-cancer activities, but the underlying mechanisms are reported to be different to different diseases [18–21]. Its anti-HSV and anti-ADV activities are through inhibition of viral replication, its anti-bladder cancer activity is through the inhibition of androgen signaling pathway, while its anti-prostate cancer activity is through the inhibition of both androgen and AKT signaling pathways [18–21]. In our study, we discovered that brassicasterol exhibited anti-HBV + HCC activity, and such activity was AKT pathway dependent. Given the depth of research on brassicasterol until now, it is hard to interpret the mechanism of action for brassicasterol’s broad anti-viral and anti-cancer activities. It is warranted, however, for future studies to further investigate whether a single target by brassicasterol resulted in such anti-viral and anti-cancer activities effecting via various downstream pathways.

AKT, a serine/threonine kinase, serves as a central node for many signaling pathways and plays an important role in cell survival, proliferation, migration, metabolism and angiogenesis [30]. AKT pathway is frequently dysregulated in many types of cancers[31]. AKT itself is also considered an oncogene, and its overexpression and over-activation are two of the major events detected in various cancers [32, 33]. Therefore, AKT has been investigated as a therapeutic target for treatment of various cancers. AKT was targeted by brassicasterol in HBV + HCC in our current study and in prostate cancer in a previous study [19]. Although it is beyond the scope of our current study, it worth to investigate if brassicasterol could inhibit the growth of other types of cancers and whether targeting AKT pathway was the underlying mechanism.

In addition to in vitro testing on cell lines, we have also confirmed the anti-HCC activity of brassicasterol on a xenograft mouse model, and consistent results were obtained from both in vitro and mouse study. Because this cell ectopic xenograft model is easy to establish at low cost and can also generate reliable results rapidly, it is a compelling model for solid cancer research, especially for anticancer drug screening [9, 29, 34]. Although being one of the widely used models in HCC research, the xenograft mouse model has its limitations [34]. Because cancer cells are injected into nude mice subcutaneously, the xenograft model fails to reflect the dynamic process of tumor-immune surveillance (34). Although beyond the scope of our current study, it would be warranted to confirm the anti-HCC activity on another animal model with intact animal or humanized immune system, before moving the study into clinical settings.

## Conclusion

Our study has shown that brassicasterol has anti-cancer activity against HBV + HCC and such activity is stronger than the FDA-approved drug sorafenib. Brassicasterol inhibits the growth of HCC through the suppression of AKT signaling pathway and the inhibitory effect on HCC is independent of HBV infection.

## Supplementary Information

**Additional file 1**. Supplementary materials.

## Data Availability

The raw data supporting the conclusions of this manuscript will be made available by the authors, without undue reservation, to any qualified researcher.

## Abbreviations

HBV: Hepatitis B virus
HCC: Hepatocellular carcinoma
