# Supplementary material for: Brassicasterol inhibits hepatitis B virus-associated hepatocellular carcinoma development via suppression of AKT signaling pathway
Source: Infect Agent Cancer. 2023 Apr 20;18:22. doi: 10.1186/s13027-023-00502-1 (PMC10116783; doi:10.1186/s13027-023-00502-1)
Supplement: Supplementary file 1 — Additional file 1. Supplementary materials. [file 13027_2023_502_MOESM1_ESM.docx]

**Supplementary materials**


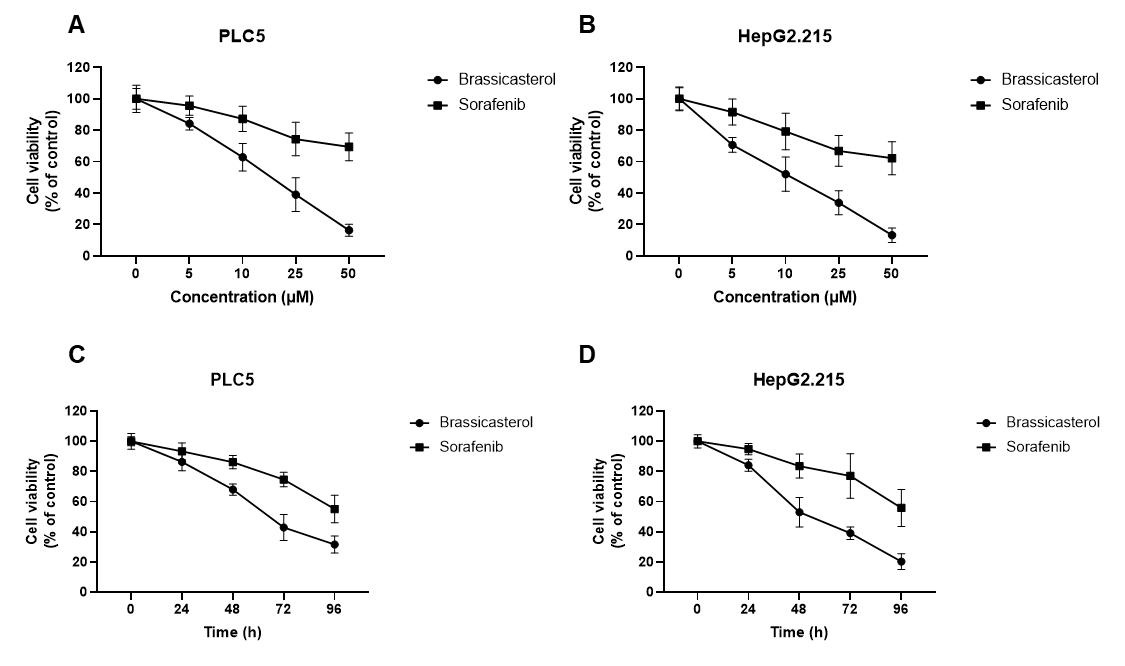


Figure S1. Brassicasterol reduces HBV+ HCC cell viability. (A) PLC5 and (B) HepG2.215 cells were incubated with increasing doses of brassicasterol or sorafenib for 48 h and cell viability was assessed by MTT assay. (C) PLC5 and (D) HepG2.215 cells were incubated with 10 µM brassicasterol or sorafenib for up to 96 h and cell viability was assessed by MTT assay. Data shown are mean +/- SD of three independent experiments.


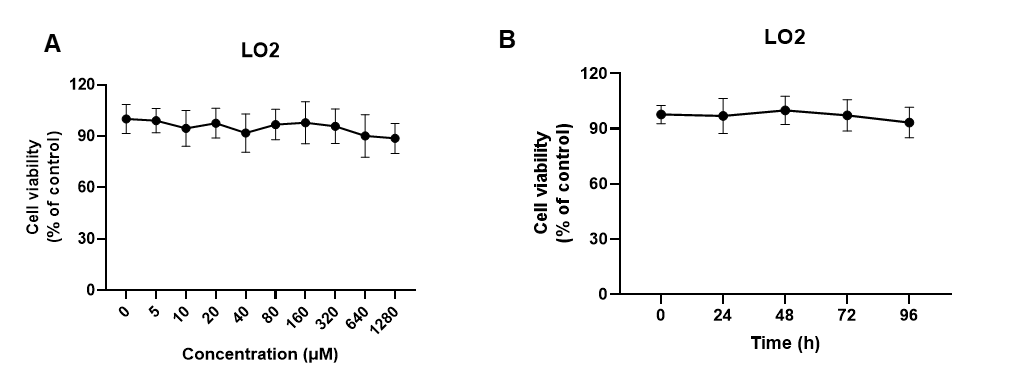


Figure S2. Brassicasterol has minimal impact on the viability of normal liver cells. (A) Normal liver cell line LO2 cells were first incubated with various doses of brassicasterol for 48 h and then cell cytotoxicity was assessed by MTT assay. (B) Cells were incubated with 1000 µM brassicasterol for up to 96 h and cell cytotoxicity was assessed by MTT assay. Data shown are mean +/- SD of three independent experiments.
